# Transcriptional benchmark dose modeling of ultraviolet radiation‐induced genomic activation in mouse skin

**DOI:** 10.1111/php.70059

**Published:** 2025-11-24

**Authors:** Sami S. Qutob, Samantha P. M. Roesch, Sandy Smiley, Pascale V. Bellier, Andrew Williams, Kate B. Cook, Matthew J. Meier, Andrea Rowan‐Carroll, Carole L. Yauk, James P. McNamee, Vinita Chauhan

**Affiliations:** ^1^ Consumer and Clinical Radiation Protection Bureau, Health Canada Ottawa Ontario Canada; ^2^ Environmental Health Science and Research Bureau, Health Canada Ottawa Ontario Canada; ^3^ Department of Biology University of Ottawa Ottawa Ontario Canada

**Keywords:** DNA damage, dose‐dependent changes, in vivo, lowest consistent response doses, oxidative stress, skin cancer, tanning, transcriptional changes, transcriptomic point of departure determination, ultraviolet radiation

## Abstract

The in vivo transcriptional response of mouse skin to ultraviolet radiation (UV‐R) exposure reveals key genomic alterations associated with UV‐R‐induced damage but it does not provide precise dose thresholds for these effects. These initial findings provided the impetus to advance dose–response characterization by integrating benchmark dose (BMD) modeling with transcriptomic data, aiming to identify biologically relevant points of departure for gene and pathway activation. To accomplish this, mice were exposed to five erythemally weighted UV‐R doses (0–40 mJ/cm^2^) emitted from a UV‐emitting tanning device, across six post‐exposure timepoints (0–96 h). Four analytical methods were used to estimate BMDs, with the lowest consistent response dose (LCRD) approach yielding the most sensitive estimates (1.21–3.44 mJ/cm^2^). Transcriptomic responses revealed activation of shared pathways related to DNA damage and cancer, oxidative stress and metabolism, inflammation and immunity, and hormonal disruption. Notably, the majority of LCRD BMD estimates (1.21–3.44 mJ/cm^2^) were lower than the International Electrotechnical Commission standard actinic exposure limit (3 mJ/cm^2^ (erythemally weighted)) for broadband UV‐R (200–400 nm) for unprotected skin and the eye for an 8 h period. These findings suggest that transcriptomic BMD modeling can detect early biological responses to UV‐R at doses lower than current exposure limits.

## Graphical abstract

Exposure to ultraviolet radiation (UV‐R) from tanning equipment increases skin cancer risk through molecular changes, but genomic thresholds remain unclear. Using mice, we examined dose‐ and time‐dependent gene expression after erythemally‐weighted UV‐R doses (0–40 mJ/cm^2^). The highest differentially expressed gene counts occurred at 6 and 48 hours in the highest dose groups. Benchmark dose (BMD) modeling using four methods identified the lowest consistent response dose (LCRD) range of 1.21–3.44 mJ/cm^2^, indicating transcriptomic changes at low doses. Median BMD analysis revealed activation of pathways linked to DNA damage, cancer, oxidative stress, inflammation, immunity, and hormonal disruption across multiple timepoints.

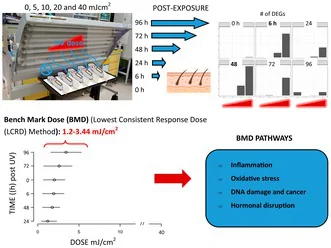

**Abbreviations**
AOPs: Adverse outcome pathways
BMD: Benchmark dose
BMDL: Benchmark dose lower confidence limit
BMDU: Benchmark dose upper confidence limit
BMR: Benchmark response
CPM: Counts per minute
CRGB: Consistent response group of BMDs
DEGs: Differentially expressed genes
GLMM: Generalized linear mixed model
IEC: International Electrotechnical Commission
IPA: Ingenuity pathway analysis
KEGG: Kyoto encyclopedia of genes and genome
LCRD: Lowest consistent response dose
LM: Linear models
MED: Minimal erythema dose
NTP: National Toxicology Program
PoD: Points of departure
PPAR: Peroxisome proliferator–activated receptors
R‐ODAF: Regulatory omics data analysis framework
ROS: Reactive oxygen species
tPoD: Transcriptomic point of departure determination
UV‐R: Ultraviolet radiation

## INTRODUCTION

It is estimated that more than a quarter of all melanomas in Canada are caused by ultraviolet radiation (UV‐R) exposure from intentional sunbathing and indoor tanning.^1^, ^2^ To better understand the mechanistic effects, numerous studies have investigated transcriptional responses in cells and tissues following UV‐R exposure. The gene expression changes observed after UV‐R exposure typically involve the activation of damage‐response pathways, which have been linked to both short‐ and long‐term adverse health effects. However, translating transcriptional data, which includes dose‐ and time‐dependent differentially expressed genes (DEGs), into human risk has proven challenging. Specifically, determining the doses needed to trigger damage‐response pathways that contribute to disease development remains elusive.

Benchmark dose (BMD) analysis estimates the dose at which a predefined increase in a biological or toxicological endpoint occurs, known as the benchmark response (BMR), providing a quantitative basis for risk assessment.^3^, ^4^, ^5^ Similarly, the definition of the transcriptomic point of departure (tPoD) is defined as the lowest dose at which a statistically significant and biologically meaningful change in gene expression is detected, based on modeling of transcriptomic dose–response data.^6^ Originally, BMDs were developed for apical toxicological endpoints, such as tumor incidence, but it has increasingly been applied to transcriptomic data, which reflect early biological changes preceding overt adverse effects. While BMD values derived from apical endpoints correspond directly to doses linked to increased risk of adverse outcomes, BMD estimates from molecular endpoints serve as PoD indicating doses at which significant biological perturbations begin to occur. The reliability of a BMD estimate is determined by the span between its upper (BMDU) and lower (BMDL) 95% confidence limits, with the BMDL frequently serving as the basis for establishing reference doses in chemical risk assessment.^7^, ^8^ These molecular points of departure can provide valuable insight into dose–response relationships and inform risk assessment by identifying sensitive biological processes.

BMD modeling has been successfully applied to characterize dose‐dependent transcriptomic responses in response to ionizing radiation.^9^, ^10^, ^11^ For UV‐R exposure, a previous study by our laboratory^12^ investigated transcriptomic and pathway expression in human epidermal keratinocytes after exposure to different doses of solar simulated UV‐R. BMD analysis identified two doses that caused significant changes in gene expression and pathways. At approximately 3 mJ/cm^2^, changes in gene expression were observed in probes and pathways related to cell signaling and DNA damage response at both 6 and 24 h postexposure. At a higher dose, near 18 mJ/cm^2^, additional transcriptional activity was associated with pathways involved in DNA repair, cellular regulation, and cancer‐related processes. The study revealed that UV‐R exposure activates DNA damage response pathways even at extremely low doses, below the limits established by international safety standards. Specifically, DNA damage response signaling was observed at doses as low as 3 mJ/cm^2^ (erythemally weighted), aligning with the actinic exposure limit for broadband UV‐R (200–400 nm) to unprotected skin and eyes over an 8‐h period, as defined by the International Electrotechnical Commission (IEC) in its IEC‐62471 (2006) standard on Photobiological safety of lamps and lamp systems.

Another recent study by our lab explored transcriptomic and pathway changes in the skin of C57BL/6N mice exposed to five erythemally weighted UV‐R doses (0, 5, 10, 20, and 40 mJ/cm^2^) emitted by a canopy sunbed.^13^ The transcriptional data from this in vivo study provided mechanistic insights into the short‐ and potential long‐term health impacts of UV‐R exposure, though it did not establish the lowest dose required to initiate these effects beyond the selected dose regimen.

To address this limitation, the current analysis aimed to determine the lowest dose for the activation of key molecular pathways involved in cancer induction due to UV‐R exposure from tanning equipment. Briefly, the previous study irradiated mouse dorsal skin with a range of UV‐R doses using a type‐2 commercial tanning canopy. Transcriptomic profiling was conducted to assess gene expression dynamics and identify activation patterns of canonical signaling pathways up to 96 h post UV‐R exposure, allowing for temporal mapping of the transcriptional response. An overarching objective of this study is to demonstrate the feasibility and interpretive value of applying dose–response modeling to next‐generation transcriptomic data. By integrating quantitative BMD modeling across multiple post‐exposure timepoints, we establish a framework for dose selection, timing, and pathway prioritization that can guide subsequent hypothesis‐driven experiments.

## MATERIALS AND METHODS

The materials and methods, including UV‐R exposure parameters, are described in the experimental protocol of our previous publication.^13^

### Bioinformatics and statistical analysis

Briefly, of the initial 150 animals, two samples were excluded due to insufficient total RNA yield (20 mJ/cm^2^—48 h and 40 mJ/cm^2^—96 h) and three additional samples from the 96 h timepoint (one each from the 5, 10, and 20 mJ/cm^2^ dose groups) were excluded due to mortality prior to collection. This resulted in 145 total RNA samples available for differential gene expression analysis. Additional sample omissions and statistical filtering methods were previously detailed in Qutob et al. (2024).^13^ The final data set comprised 142 samples, with a minimum of four replicates per experimental group.

A count matrix comprising these sequenced samples was imported into R for analysis. Differential expression was assessed using DESeq2 v1.36.1^14^ with log_2_ fold change shrinkage via ashr. Results were extracted using an alpha of 0.1 (Wald test *p*‐value), and false discovery rate (FDR)‐adjusted *p*‐values were reported. Cook's distance filtering was disabled (cutoff = FALSE).

Following the Regulatory Omics Data Analysis Framework (R‐ODAF) guidelines,^15^ genes were retained if ≥75% of samples in at least one group exceeded 1 counts per million (CPM). Spurious spikes were removed based on the condition: (max—median) < (sum of counts)/(*n* + 1). DEGs were defined by a linear fold change of ≥1.5 and an FDR‐adjusted *p*‐value of <0.05. The master DEG list (*n* = 2363) comprised genes meeting these criteria in any single condition. Common DEGs were defined as genes from this list with ≥1.5‐fold change across all timepoints or conditions, without additional filtering.

Pathway analysis (i.e. KEGG and Wikipathway functional enrichment of DEGs) was conducted using the ClusterProfiler package (Version 4.2.1) as previously detailed in Qutob et al. (2024).^13^

### Benchmark Dose (BMD) Modeling analysis

A generalized linear mixed model (GLMM) for differential gene expression was used. BMDExpress uses linear models (LM) assuming normality, where log_2_‐transformed CPM were used to meet this assumption. Prefiltering was performed by retaining probes with ≥1.5‐fold change in at least one condition and an unadjusted *p*‐value of <0.05, following National Toxicology Program (NTP) guidelines.^16^

We tested BMR values ranging from 10% to 50% and selected a BMR of 50% to balance sensitivity and specificity while ensuring biologically meaningful dose–response signals. Benchmark dose (BMD) analysis was then conducted using a BMR of 0.25%, based on relative deviation. Bayesian model averaging was applied using default model settings.^17^ Filters were applied to exclude BMD estimates if they exceeded the highest tested dose, if the BMD to Benchmark Dose Lower Confidence Limit (BMDL) ratio was >20, or if the Benchmark Dose Upper Confidence Limit (BMDU) to BMDL ratio exceeded 40.

### Derivation of in vivo tPoD

Four approaches were considered to derive a tPOD: (1) the BMD of the 25th ranked gene, (2) the lowest consistent response dose (LCRD), (3) the lowest median KEGG pathway BMD, and (4) the lowest 5th percentile KEGG pathway BMD. The 95% confidence intervals were obtained using the residual bootstrap method.^18^

Initial analysis has the 25th ranked gene, with the lowest median pathway using Kyoto Encyclopedia of Genes and Genomes (KEGG) and the lowest consistent response dose (LCRD). All genes were ranked from lowest to highest. The 25th rank‐ordered gene was set as the threshold and was used to indicate the dose where a defined change in the transcriptome has occurred.^19^, ^20^ Doses of UV‐R that were unable to produce a representative value for this tPOD (i.e., <25 responsive genes) were identified as “inactive” based on this approach and alternative methods were considered for tPOD derivation.

The LCRD was determined using the approach outlined by Crizer et al. (2021).^21^ It is defined as the lowest‐ranked gene in an ordered list where the ratio values of all subsequent genes remain within 1.66, effectively filtering out unrealistically low gene BMDs that may represent biological noise. The genes within this ranked set constitute the consistent response group of BMDs (CRGB), since each sequential BMD in this group has at least one BMD within a 1/4 log difference. The lowest BMD in the CRGB is then designated as the LCRD. However, this method can be sensitive to exceptionally low BMDs, potentially resulting in an overly conservative estimate, particularly when extrapolated below the lowest dose. To mitigate this, a modified approach is proposed, in which BMDs are grouped as described above, but the LCRD is identified as the lowest dose from the largest CRGB. This refinement prevents small groups (*n* = 2 or 3) of genes with low BMDs from being misclassified as the LCRD.

### Pathway enrichment analysis

Gene‐ and pathway‐specific BMD values were derived to establish pathway‐based PODs using gene identifiers from transcriptomic datasets analyzed in Ingenuity Pathway Analysis (IPA). Fold changes at the highest dose were applied with thresholds of ≥1.5 or ≤0.66 (*p* ≤ 0.05). Median pathway BMDL values were then calculated for canonical pathways that included at least three genes.

## RESULTS

### Benchmark dose modeling

#### Overall trends in dose–response

Dose–response curves from high‐throughput transcriptomic data were produced using the transcriptomic workflow described by Vuong et al. (2025).^22^ Genes that were modeled all passed preliminary filters establishing that they surpassed a 1.5‐fold change relative to controls and showed a dose–response trend using a Williams' trend test (*p*‐value <0.05). Each gene's BMD represents the dose at which the gene expression shows a statistically significant change relative to the baseline (no exposure) at the same timepoint, corresponding to the chosen BMR level (BMR = 0.25). The number of genes with BMDs and the median transcriptomic BMD and BMDL across the six timepoints are presented in Table 1.

**TABLE 1:** Summary of the gene‐level benchmark dose.

Time point	Number of genes with BMDs	Median BMD	Median BMDL
0 h	271	19.76	7.35
6 h	751	21.23	9.64
24 h	1100	20.86	9.13
48 h	2312	20.35	10.13
72 h	1001	21.08	10.09
96 h	408	19.11	8.74

The number of genes with BMD values varied over time, peaking at 48 h post‐exposure (2312 genes) at a median BMD of 20.35 mJ/cm^2^ (median BMDL value of 10.13 mJ/cm^2^), before decreasing at later timepoints. The median BMD values remained relatively consistent across timepoints, ranging from 19.11 to 21.23 mJ/cm^2^, while the median BMDL values ranged from 7.35 to 10.13 mJ/cm^2^.

#### Gene accumulation from dose response models

**FIGURE 1:**
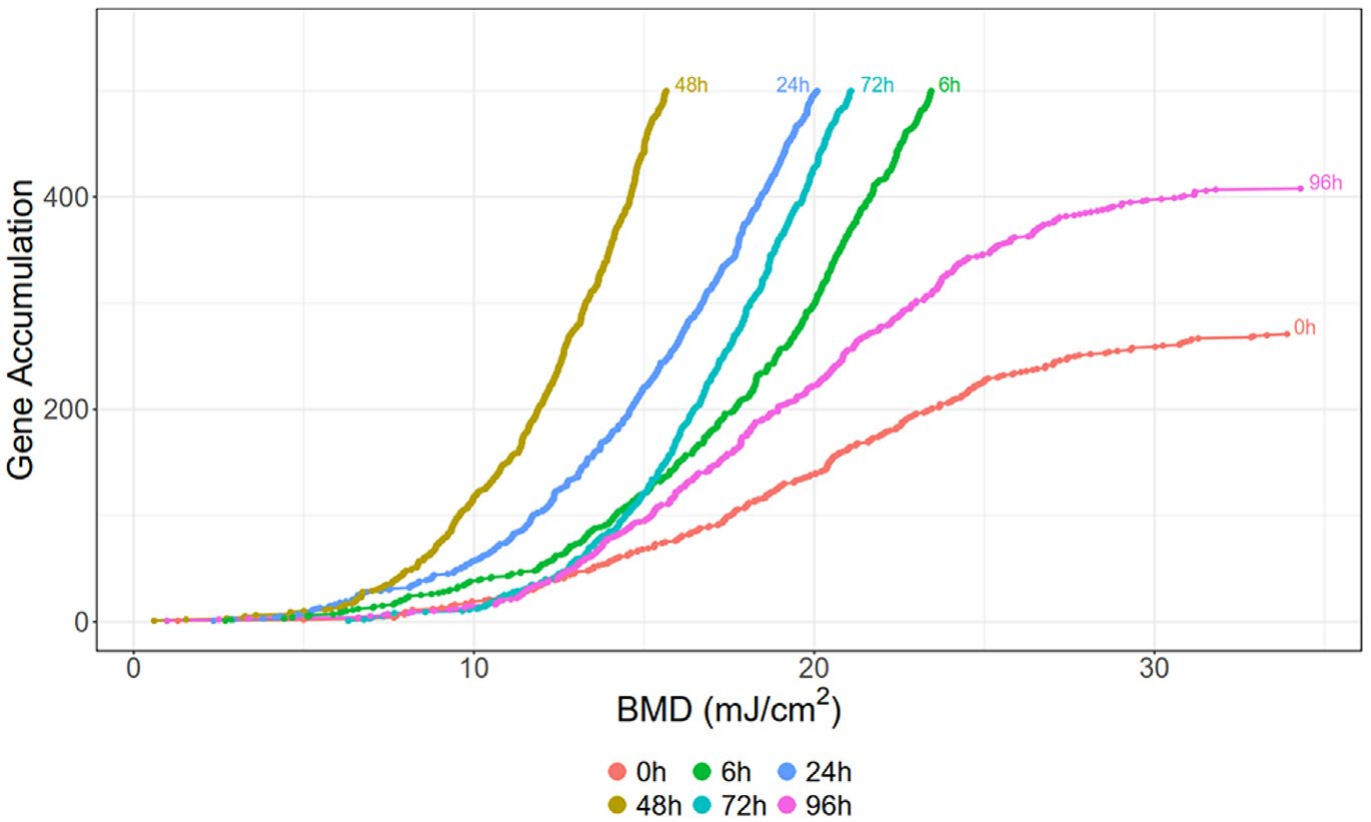
Time–response gene accumulation plots following exposure to five erythemally weighted doses of UV‐R emitted from a UV‐emitting tanning device.

Gene‐level accumulation plots illustrate the distribution of BMD values across individual genes, providing a profile of biological effects (Figure 1). In these plots, genes are rank‐ordered from lowest to highest BMD along the y‐axis, while their corresponding BMD values are shown on the x‐axis. The most sensitive genes, with the lowest BMD values, appear at the lower end of the x‐axis. A steeper curve indicates increased transcriptional activity within a narrow dose range. The overall pattern of biological activity mirrors the previously observed median gene BMD trend, with the strongest response occurring at the 48 h timepoint. In contrast, the 0 h timepoint showed the weakest response, followed by the 96 h timepoint as the second least responsive compared to the other timepoints.

**FIGURE 2:**
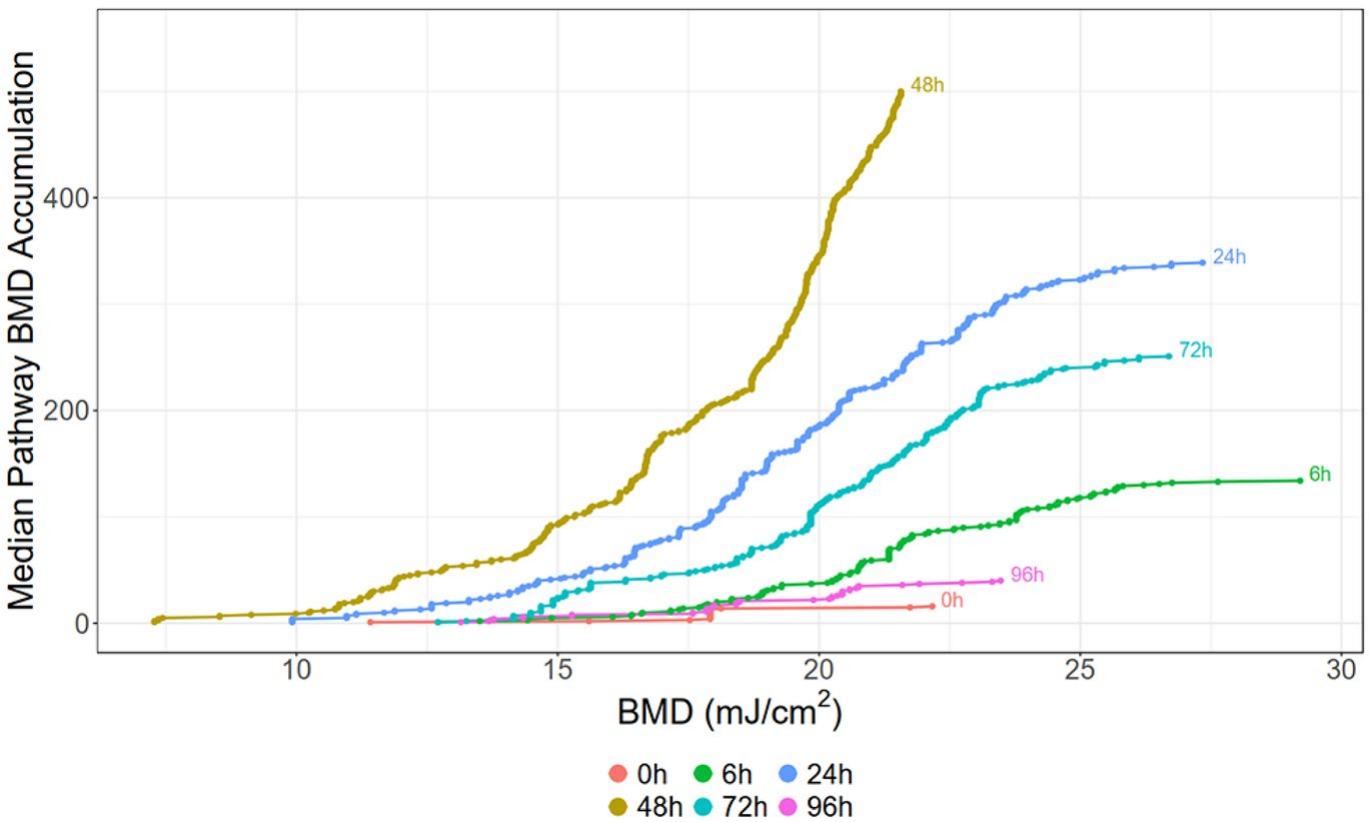
Time–response median KEGG pathway accumulation plots following exposure to five erythemally weighted doses of UV‐R emitted from a UV‐emitting tanning device.

Median Kyoto Encyclopedia of Genes and Genomes (KEGG) Pathway BMD accumulation plots (Figure 2) further summarize these trends by aggregating gene‐level BMDs into pathway‐level responses. The differences between timepoints become even more pronounced in these pathway‐level analyses yet the pattern of potency remains the same as the gene‐level accumulation plots. The 48 h timepoint remains the most potent, followed by 24 h and 72 h, whereas 6 h, 96 h, and 0 h exhibit lower transcriptional responses across all accumulation thresholds. The 0 h timepoint remains the weakest response, again closely followed by the 96 h timepoint.

#### Approaches for Identifying tPoD

**FIGURE 3:**
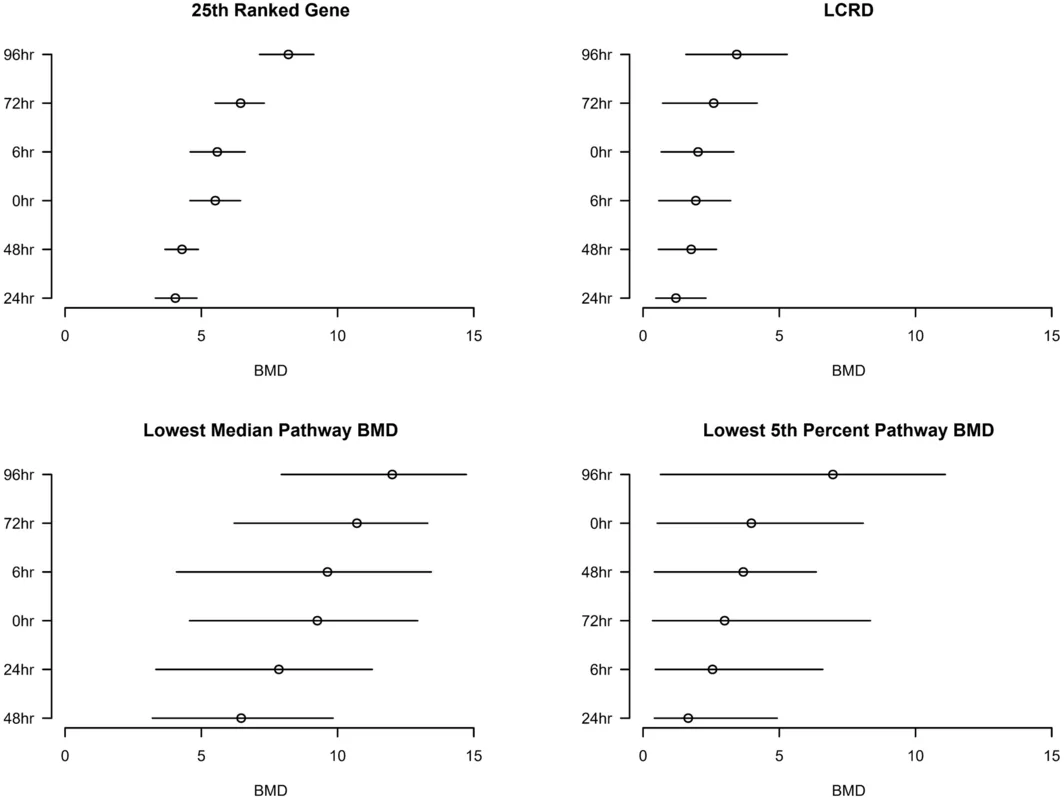
Comparison of 95% transcriptional PoD Confidence Intervals and BMDs. Four approaches were evaluated to derive a transcriptomic point of departure (PoD): (1) the benchmark dose (BMD) of the 25th ranked gene, (2) the lowest consistent response dose (LCRD), (3) the lowest median BMD among KEGG pathways, and (4) the lowest 5th percentile BMD among KEGG pathways.

The results of the four approaches of deriving tPoDs: (1) the BMD of the 25th ranked gene, (2) the lowest consistent response dose (LCRD), (3) the lowest median KEGG pathway BMD, and (4) the lowest 5th percentile KEGG pathway BMD are presented in Figure 3 and detailed in Table S1.

The confidence intervals associated with pathway‐based tPoDs were substantially broader than those observed in gene‐level approaches, aligning with findings from Addicks et al. (2023).^23^ This increased uncertainty likely stems from the aggregation of multiple gene responses within a pathway, which amplifies variability in the overall benchmark dose estimation. The LCRD consistently has the lowest BMD estimates across timepoints (range 1.21–3.44 mJ/cm^2^), suggesting it identifies transcriptomic changes at lower doses than the other methods. Whereas the lowest median KEGG pathway had the highest BMD estimates across timepoints (range 6.46–12.01 mJ/cm^2^).

While tPoD estimates from the different approaches are largely comparable, differences are observed for the 25th ranked gene, particularly across timepoints (range 4.05–8.2 mJ/cm^2^). However, all median estimates fall within approximately two standard deviations of each other, suggesting that despite these variations, the overall tPoD estimates remain relatively similar.

#### Pathway analysis

In the previous transcriptomics study,^13^ we examined commonly perturbed genes/pathways across timepoints to help delineate underlying biological mechanisms associated with UV‐R exposure. Similarly, KEGG pathway BMD summaries were generated using the median pathway BMD to identify common pathways across the six timepoints. Pathways were included in the analysis if they contained at least three genes with BMD estimates and if at least 5% of the pathway's genes had BMDs.^16^

**FIGURE 4:**
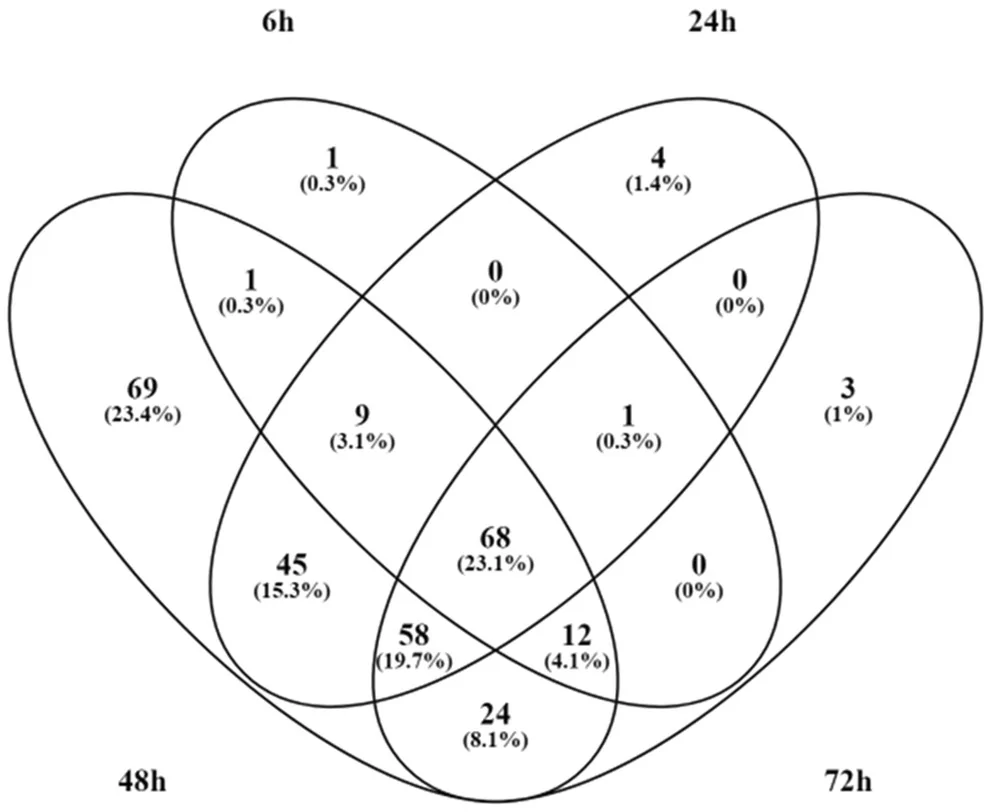
Venn diagram depicting unique and common KEGG pathways with pathway‐level BMD estimates for the 6 h, 24 h, 48 h, and 72 h timepoints.

Figure 4 presents a Venn diagram summarizing the shared and unique KEGG pathways at 6, 24, 48, and 72 h, which exhibit strong potency (sharing a minimum of 90 pathways with the most responsive 48 h timepoint). Across the four timepoints shown in Figure 4, a total of 68 common pathways were identified, which can be grouped into five major biological categories. These categories are Inflammatory and Immune Response Pathways, Metabolic Pathways, Cardiovascular and Endocrine Signaling, Nervous System and Neurological Disorders, Digestive, Excretory System Pathways, and Cancer and Cell Regulation.

In contrast, Figure S1 compares the 0, 24, 48, and 96 h timepoints, highlighting common pathways between the more potent 24 h and 48 h responses and the less responsive 0 h and 96 h timepoints. Only two pathways, mineral absorption and tyrosine metabolism, were common in Figure S1. Mineral absorption was the only pathway consistently observed across all six timepoints.

The specific KEGG pathways associated with each category, from Figure 4, are provided in Table S2. These shared six categories have been summarized as: 1. Inflammatory and Immune Response Pathways, 2. Metabolic Pathways, 3. Cardiovascular and Endocrine Signaling, 4. Cancer and Cell Regulation, 5. Nervous System and Neurological Disorders, and 6. Digestive and Excretory System Pathways. Their corresponding ranges of median BMD and BMDL values for the peak gene expression timepoint of 48 h are 1. BMD (17.93–22.66); BMDL (8.85–11.84), 2. BMD (15.74–20.95); BMDL (5.76–11.7), 3. BMD (19.82–24.52); BMDL (8.87–12.64), 4. BMD (19.5–22.39); BMDL (7.9–11.7), 5. BMD (20.43–24.46); BMDL (10.65–12.87), and 6. BMD (19.81–20.99); BMDL (10.65–10.72). Additional BMD and BMDLs of the various timepoints can be found in Table S2.

From the perspective of UV‐R exposure, these pathways can be further grouped into four key categories. These broad categories have been summarized as: 1. DNA Damage and Cancer‐Associated Pathways, 2. Oxidative Stress and Metabolic Regulation, 3. Immune and Inflammatory Response Pathways, and 4. Hormonal and Endocrine Disruption. Their corresponding ranges of median BMD and BMDL values for the peak gene expression timepoint of 48 h are 1. BMD (19.5–22.39); BMDL (9.08–11.7), 2. BMD (15.74–18.8); BMDL (5.76–11.7), 3. BMD (18.15–22.27); BMDL (8.85–12.58), and 4. BMD (19.82–24.52); BMDL (8.87–12.64). The BMD and BMDL values for the other timepoints are provided in Table S3.

## DISCUSSION

In this study, we applied a BMD approach to KEGG pathways across multiple timepoints to identify common and dose/time‐specific transcriptomic responses in mouse skin after UV‐R exposure. By integrating pathway‐level BMD estimates, we observed substantial variability in confidence intervals, consistent with previous findings.^23^ This variability likely arises from pathway‐level aggregation, where contributions from multiple genes increase uncertainty in overall BMD estimates. Nevertheless, pathway‐based BMDs continue to be valuable for identifying biologically meaningful dose‐dependent responses, as aggregating gene‐level data into pathways enables the linkage of molecular changes to established biological functions. Four approaches were evaluated to derive transcriptomic PoDs. Among them, the LCRD consistently produced the lowest BMD estimates across timepoints, indicating its sensitivity in detecting transcriptomic changes at lower doses. To address concerns regarding the sensitivity of the LCRD method to outliers and its tendency to exclude highly variable or low‐response genes, we employed a modified approach. Specifically, we selected the lowest dose from the largest Consistent Response Gene Group (CRGB), rather than relying on the smallest numerical benchmark dose (BMD) cluster. This adjustment minimizes bias introduced by small gene sets with low BMDs and enhances the robustness of the threshold estimation.

Model reliability was further ensured by applying standard quality filters, excluding fits where BMD exceeded the maximum tested dose, BMD/BMDL ratios exceeded 20, or BMDU/BMDL ratios exceeded 40. Additionally, 95% confidence intervals were derived using bootstrap resampling to account for uncertainty.

The resulting LCRD thresholds (1.2–3.4 mJ/cm^2^) were consistent with other tPoDs, including the 25th ranked gene and pathway‐based BMDs. These findings support the interpretation that LCRD values provide a conservative and biologically meaningful estimate of the lower bound for transcriptional activation, rather than an artifact of outlier influence. tPoD estimates were generally comparable across methods, suggesting minimal variation in the pathway's lowest dose of activation. While BMDs based on the 25th‐ranked gene showed some variability, median values across approaches remained within a two‐fold range, highlighting the overall robustness of the dose–response estimates.

In our previous transcriptomic study,^13^ it was determined that the 40 mJ/cm^2^ dose was the minimal erythema dose (MED) for C57BL/6 N mice, 48 h post‐exposure. Interestingly, it was observed that even a low UV‐R dose of 5 mJ/cm^2^, equivalent to just 12.5% of 1 MED, induced significant transcriptomic changes, with 116 DEG detected at 96 h post‐exposure. These changes were associated with structural changes, metabolism, immune response, apoptosis, and mitochondrial function from UV‐R damage. However, determination of transcriptional PoDs extrapolated even lower dose values for significant differential gene expression. The LCRD method yielded the lowest tPoDs, ranging from 1.21 to 3.44 mJ/cm^2^, with a value of 1.77 mJ/cm^2^ at the 48‐h timepoint. In comparison, the 25th‐ranked gene approach produced slightly higher PoDs, between 4.05 and 8.2 mJ/cm^2^ (4.29 mJ/cm^2^ at 48 h), yet these values remained well below the BMDL of 10.13 mJ/cm^2^ observed at 48 h post‐exposure. Notably, the lowest transcriptional PoD value of 1.21 mJ/cm^2^ represents approximately only 3% of the 1 MED of 40 mJ/cm^2^. A previous study in hairless mice found that as little as 10–20% of the MED was sufficient to observe mutations.^24^ The significantly lower threshold value (3% of 1 MED) in the current study demonstrates the usefulness of utilizing BMD analysis in determining genomic sensitivity, which is substantially lower than traditional methods of determining thresholds of UV‐R responsiveness.

The number of genes with BMD values (BMR = 0.25) peaked at 48 h post‐exposure, suggesting that 48 h may represent a critical window for UV‐R‐induced gene regulation and associated biological processes. The median transcriptomic BMD at this timepoint was 20.35 mJ/cm^2^, a dose that is half that required to cause erythema in mice at the same timepoint. Consistent with this, our earlier study of differential gene expression (*p*_adj_ <0.05 and |FoldChange| > 1.5) found the most significant transcriptional changes (both in the number of DEGs and the magnitude of fold change), at the 6‐ and 48‐h timepoints after exposure to 20 and 40 mJ/cm^2^, relative to their time‐matched sham controls.^13^ It is clear that BMD analysis values are in line with both physiological and transcriptional expression changes. Functional analysis at 48 h revealed that both the 20 and 40 mJ/cm^2^ doses caused commonly enriched pathways related to oxidative phosphorylation, DNA replication, cell cycle regulation, PI3K‐AKT, JAK–STAT, and AMPK signaling, thermogenesis, and insulin and glucagon signaling. Notably, strong activation of the p53 signaling pathway at the 48‐h timepoint suggested a robust cellular response to DNA damage and the initiation of repair mechanisms at both dose levels.^13^

In chemical toxicology, conservative acceptable limits for reliable risk assessment are typically based on BMDL rather than BMD values.^25^ In this study, the number of genes peaked at 48 h post‐exposure at a BMDL value of 10.13 mJ/cm^2^. This represents the lower limit of the 95% confidence interval for the BMD, indicating the lowest dose that could still produce the observed substantial change in gene expression. This suggests that doses below approximately 10 mJ/cm^2^ are unlikely to trigger the same adverse gene expression changes; however, transcriptional PoD estimates indicated that a more conservative threshold dose is ~1 mJ/cm^2^.

Median BMDs revealed that common pathways associated with UV‐R exposure at the 6, 24, 48, and 72 h timepoints could be classified into four key mechanistic categories:

1. DNA Damage and Cancer‐Related Pathways (e.g., p53 signaling, apoptosis, transcriptional misregulation in cancer): This category reflects the well‐established role of UV‐R as an environmental carcinogen. UV‐R induces direct DNA damage, promotes mutation accumulation, and activates apoptotic pathways. These mechanisms have been implicated in the pathogenesis of skin cancers such as melanoma and squamous cell carcinoma.^26^ Peak expression at 48 h post‐exposure, the BMDL range values of this pathway were 9.08–11.7 mJ/cm^2^ comparable to the PoD BMDL value of 10.13 mJ/cm^2^.
2. Oxidative Stress and Metabolic Pathways (e.g., glutathione metabolism, arachidonic acid metabolism, cytochrome P450 pathways): UV‐R exposure leads to the generation of reactive oxygen species (ROS), which in turn cause lipid peroxidation, protein oxidation, and DNA damage.^27^ The glutathione metabolism pathway is particularly important for mitigating oxidative stress through detoxification of ROS, while cytochrome P450 enzymes and lipid metabolism pathways are involved in cellular homeostasis.^27^, ^28^ At 48 h post‐exposure, the BMDL range values of this pathway were 5.76–11.7 mJ/cm^2^.
3. Inflammatory and Immune Response Pathways (e.g., IL‐17, TNF, NF‐κB, NOD‐like receptor signaling): UV‐R exposure modulates both innate and adaptive immune responses, leading to inflammation, immunosuppression, and increased susceptibility to skin infections and autoimmune conditions.^29^ Key pathways such as NF‐κB, TNF, and IL‐17 signaling are central mediators of UV‐induced inflammatory responses and were also found in another transcriptomic study examining responses to UV‐A and UV‐B radiation in human keratinocytes.^30^ At 48 h post‐exposure, the BMDL values for this pathway ranged from 8.85 to 12.58 mJ/cm^2^.
4. Hormonal and Endocrine Disruption Pathways (e.g., PPAR signaling, thyroid hormone synthesis, calcium signaling): UV‐R may influence hormonal regulation by affecting endocrine‐related signaling pathways.^31^ For example, peroxisome proliferator–activated receptors (PPAR) signaling is involved in lipid metabolism and the maintenance of skin barrier integrity, and its dysregulation may contribute to UV‐R‐induced alterations in endocrine function.^32^, ^33^ Last, similar to the previously noted pathway at 48 h, the BMDL values ranged from 8.87 to 12.67 mJ/cm^2^. These values from the four common pathways associated with UV‐R exposure suggest that doses below approximately 10 mJ/cm^2^ are unlikely to induce the same adverse pathway alterations.

Results from a previous study by our group provide an opportunity for direct comparison of BMD analyses, though several key methodological differences must be considered.^12^ The 2018 study utilized global gene expression profiling via Illumina microarray technology in cultured human keratinocytes exposed to three doses of solar‐simulated UV‐R (matched to mid‐July sunlight in Ottawa, Ontario, Canada), with gene expression measured only at 6 and 24 h post‐exposure. BMD analysis followed traditional methods, applying the lowest 10th percentile, a benchmark response (BMR) of 10%. In contrast, the current study involved in vivo UV‐R exposure of mouse skin using four incrementally spaced doses from a commercial tanning bed, followed by tissue extraction and transcriptomic profiling at 0, 6, 24, 48, 72, and 96 h post‐exposure. Gene expression was assessed via Next‐Generation RNA‐Seq using total RNA, with preprocessing aligned to the R‐ODAF. BMD modeling in this study employed a BMR of 25%, chosen to reflect the one standard deviation approach recommended by the U.S. EPA^34^ and based on the work by Zeller et al. (2017)^35^ in selecting the appropriate critical effect size. Lower (10%) and higher (50%) BMRs were found to be either overly restrictive, excluding biologically relevant UV‐R–responsive genes or overly inclusive, capturing nonspecific gene responses. Additional approaches were also applied, including the 25th‐ranked gene BMD derived from gene accumulation plots. Despite methodological differences between the studies, including species, exposure sources, timepoints, analytical techniques, and BMD threshold values, concordant findings emerged. Notably, the axon guidance pathway was consistently identified at approximately 20 mJ/cm^2^, 6 h post‐exposure, in both the in vitro and in vivo models.

Interestingly, the 2018 study showed a unique bimodal BMD response with peaks at 2.72 mJ/cm^2^ and 18.4 mJ/cm^2^ following exposure to environmentally relevant UV‐R emitted from a solar simulator in human epidermal keratinocytes.^12^ The higher BMD value (Mode 2) of 18.4 mJ/cm^2^ (erythemally weighted) closely corresponds to the 20 mJ/cm^2^ dose known to induce minimal erythema in individuals with Type I fair skin. This BMD is linked to pathways involved in cell signaling, cell cycle control, DNA repair, and oncogenic processes. The median transcriptomic BMD of 20.35 mJ/cm^2^ in the current study aligns with the in vitro findings and physiological outcomes, reinforcing their relevance.

The lower BMD value (Mode 1) from our in vitro study was at a dose of 2.72 mJ/cm^2^ (erythemally weighted), 24 h post‐exposure. This dose closely approximated the actinic exposure limit of 3 mJ/cm^2^ (erythemally weighted over an 8‐h period) established in IEC 62471:2006. Notably, the 2.72 mJ/cm^2^ dose was associated with the activation of pathways related to nucleotide biosynthesis and cell cycle regulation. In the current study, tPODs of 4.29 (25th Ranked Gene) and 1.77 (LCRD) were identified at the 48 h timepoint. While the BMD values are similar, it must be noted that the erythema threshold in mouse skin differs slightly from humans, appearing at 48 h post‐exposure rather than 24 h.^36^ Regardless, the monomodal BMD values in the current study are consistent with the BMD values previously identified in human keratinocytes^12^ and align with current human UV‐R exposure limits and the minimal erythemal dose.

While our study is exploratory in nature, we also note that the transcriptomic data align with established UV‐R mechanisms, including DNA damage response, oxidative stress, and inflammatory signaling, and corroborate previous in vitro and in vivo findings. This concordance underscores the biological relevance of the results and highlights the potential of the tPOD framework to guide future mechanistic and biomarker‐driven research.

## CONCLUSION

These findings highlight the utility of transcriptomic BMD approaches as a valuable complement to traditional toxicological assessments by identifying biologically relevant, pathway‐level dose thresholds. This study demonstrates that gene/pathway‐based BMD modeling can consistently capture meaningful dose–response relationships across multiple timepoints, with recurrent enrichment of pathways associated with DNA damage, oxidative stress, inflammation, and endocrine disruption. Collectively, these results provide a mechanistic framework for understanding UV‐R‐induced effects and underscore the potential role of transcriptomic BMDs in risk assessment and regulatory decision‐making. Moreover, these findings offer an opportunity for further alignment with Adverse Outcome Pathways (AOPs) but would be strengthened with BMD analysis of DEG responses in human skin following UV‐R exposure.

## AUTHOR CONTRIBUTIONS

SQ, SR, PB, and JM conceptualized and designed the study. SQ, JM, PB, SR, and SS conducted the animal experiments, and SQ, SR, and SS isolated RNA samples. ARC conducted the RNASeq analysis. AW, KC, and MM performed the bioinformatics and statistical analysis. SQ wrote the manuscript with significant intellectual input from JM and VC. This study was led by SQ, who held the role of principal investigator.

## Supporting information


Figure S1.

Table S1.

Table S2.

Table S3.

## Data Availability

The data that support the findings of this study are available from the corresponding author upon reasonable request.
